# Functional characterization of cohesin subunit SCC1 in *Trypanosoma brucei* and dissection of mutant phenotypes in two life cycle stages

**DOI:** 10.1111/j.1365-2958.2008.06320.x

**Published:** 2008-06-16

**Authors:** Eva Gluenz, Reuben Sharma, Mark Carrington, Keith Gull

**Affiliations:** 1 Sir William Dunn School of Pathology, University of OxfordSouth Parks Road, Oxford OX1 3RE, UK; 2 Department of Biochemistry, University of Cambridge80 Tennis Court Road, Cambridge CB2 1GA, UK

## Abstract

In yeast and metazoa, structural maintenance of chromosome (SMC) complexes play key roles in chromosome segregation, architecture and DNA repair. The main function of the cohesin complex is to hold replicated sister chromatids together until segregation at anaphase, which is dependent on proteolytic cleavage of the cohesin subunit SCC1. Analysis of trypanosomatid genomes showed that the core cohesin and condensin complexes are conserved, but SMC5/6 is absent. To investigate the functional conservation of cohesin in eukaryotes distantly related to yeast and metazoa, we characterized the *Trypanosoma brucei* SCC1 orthologue. TbSCC1 is expressed prior to DNA synthesis at late G1, remains in the nucleus throughout S- and G2-phases of the cell cycle and disappears at anaphase. Depletion of SCC1 by RNAi or expression of a non-cleavable SCC1 resulted in karyokinesis failure. Using the dominant negative phenotype of non-cleavable SCC1 we investigated checkpoint regulation of cytokinesis in response to mitosis failure at anaphase. In the absence of chromosome segregation, procyclic trypanosomes progressed through cytokinesis to produce one nucleated and one anucleate cell (zoid). In contrast, cytokinesis was incomplete in bloodstream forms, where cleavage was initiated but cells failed to progress to abscission. Kinetoplast duplication was uninterrupted resulting in cells with multiple kinetoplasts and flagella.

## Introduction

The conserved structures of chromatin, chromosomes and the nucleus are defining features of eukaryotic cells. Trypanosomatids belong to a eukaryotic supergroup (Simpson and Roger, 2004; Adl *et al*., 2005) separate from fungi/animals or plants. Their chromatin is based on nucleosomes containing canonical core histones H2A, H2B, H3 and H4 (Hecker *et al*., 1994) although the linker histone H1 is atypical, lacking a globular N-terminal domain (Alsford and Horn, 2004). Higher-order chromatin structure remains uncharacterized but it is known that condensation to 30 nm fibres does not occur (Hecker *et al*., 1994) and there is no visible condensation of chromosomes during mitosis.

In *Trypanosoma brucei*, the chromosomes contain a single linear DNA molecule and there are 11 diploid pairs of ‘large’ chromosomes from 1 to 6 Mb. In addition there are several intermediate chromosomes, from 200 to 900 kb, and ∼100 minichromosomes, from 50 to 150 kb (Ersfeld *et al*., 1999). The ploidy of these smaller chromosomes is uncertain. Other trypanosomatid species such as *Trypanosoma cruzi* (Gibson and Miles, 1986) or *Leishmania* (Ivens *et al*., 2005) do not contain the two categories of smaller chromosome and in *T. brucei* the smaller chromosomes are probably an adaptation to increase the number of telomeres which act as favoured substrates in the gene conversion-based system of antigenic variation (Robinson *et al*., 1999). The functional organization of the genome of *T. brucei,* and other Trypanosomatida, is unique (Berriman *et al*., 2005). On the large chromosomes, genes are arranged in polycistronic transcription units of tens of genes that are transcribed constitutively by RNA polymerase II. Monocistronic mRNAs are produced by post-transcriptional processing (Palenchar and Bellofatto, 2006). Putative discrete centromeric DNA sequences in *T. brucei* large chromosomes were recently identified by mapping topoisomerase II cleavage sites (Obado *et al*., 2007).

The behaviour of chromosomes during mitosis has been investigated in *T. brucei* where mitosis is closed without breakdown of the nuclear envelope (Ogbadoyi *et al*., 2000). There is an intranuclear spindle (Ersfeld and Gull, 1997) and some spindle microtubules appear to terminate in trilaminar kinetochore-like structures (Ogbadoyi *et al*., 2000). Segregation of chromosomes occurs in association with the spindle but large and mini-chromosomes segregate with different dynamics (Ersfeld and Gull, 1997; Gull *et al*., 1998). The total number of chromosomes exceeds that of spindle microtubules and kinetochores so the segregation of smaller chromosomes is not straightforward. A current model proposes that the small chromosomes form a lateral association with antiparallel microtubules in the central area of the pole-to-pole spindle and are transported along the spindle microtubules by motor proteins (Gull *et al*., 1998). The 11 large chromosomes by contrast are thought to attach to microtubules via kinetochores and their segregation mechanism is more similar to that of yeast chromosomes.

In yeast and metazoa, sister chromatid cohesion from S-phase to anaphase is maintained by cohesin, a structural maintenance of chromosome (SMC) complex (Losada and Hirano, 2005; Nasmyth and Haering, 2005). SMC complexes are composed of a SMC heterodimer, a kleisin and specific non-SMC subunits. In the case of cohesin, the α-kleisin is SCC1 which together with SMC1 and SMC3 forms a tripartite ring structure necessary for sister chromatid cohesion (Gruber *et al*., 2003). The fourth component of cohesin is the STAG domain protein SCC3. A second type of SMC complex, condensin, has a role in higher-order chromatin structure and contains SMC2 and SMC4, along with a β- or γ-kleisin and HEAT repeat proteins (Nasmyth and Haering, 2005).

The separation of sister chromatids prior to cell division is dependent on a chain of events starting with the activation of the anaphase-promoting complex (APC), an E3 ubiquitin ligase. One of the first proteins ubiquitinylated by the APC, and subsequently proteolysed, is securin which is a stoichiometric inhibitor of the proteolytic activity of separase (Ciosk *et al*., 1998). One substrate of separase is the cohesin subunit SCC1 which is cut at one or both of two specific sites, an event both necessary and sufficient for the onset of anaphase A (Uhlmann *et al*., 1999; Wirth *et al*., 2006).

Yeast and metazoa represent a small fraction of eukaryotic diversity. Here, we provide evidence that orthologues of cohesin and condensin components are present in trypanosomatids and that basic mechanisms of sister chromatid cohesion are conserved and thus evolved early. A functional analysis of *T. brucei* SCC1 was performed and depletion of SCC1 decreased the growth rate and resulted in nuclear division defects without arresting the cell cycle. Expression of a separase-resistant mutant of SCC1 was used to produce a dominant negative phenotype with cells unable to separate sister chromatids and thus arrested in early anaphase. The cellular phenotype of the anaphase arrest was different in the two life cycle stages investigated. Procyclic-form cells completed cytokinesis in the absence of mitosis resulting in production of anucleate cells with one kinetoplast (zoids) whereas bloodstream forms initiated cytokinesis without progressing to abscission.

## Results

### SMC complexes in trypanosomes – the mitotic cohesin complex is conserved

**Fig. 1:**
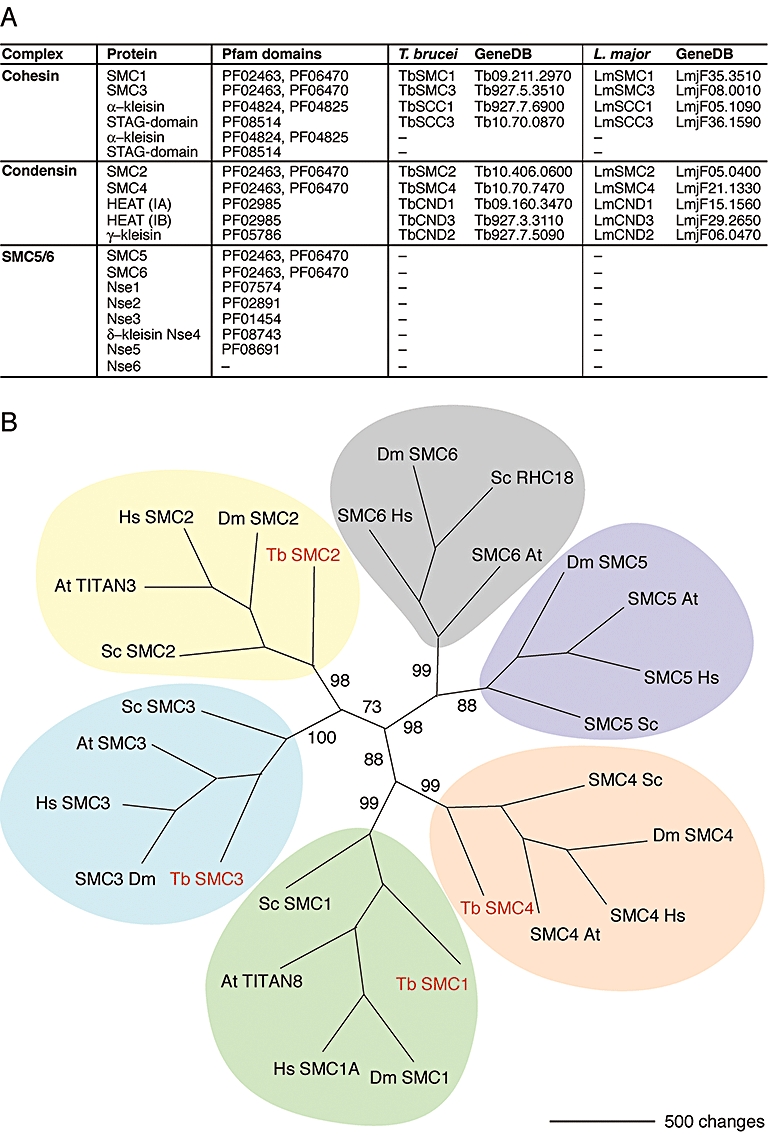
A. Orthologues of SMC1–4 and non-SMC subunits of the cohesin and condensin complex were identified in the genomes of *Trypanosoma brucei* and *Leishmania major* by (i) hmmer searches using seed alignments from Pfam and (ii) ‘reciprocal best blast hits’ for the corresponding proteins from yeast. The accession numbers for GeneDB (http://www.genedb.org) and Pfam domains (http://pfam.sanger.ac.uk/) are listed. B. Phylogenetic analysis was used to classify the trypanosome SMC proteins. Kinetoplastid genomes encode four SMC proteins that are orthologues of SMC1–4. A maximum parsimony phylogram was built using sequence alignments of the four *T. brucei* SMC sequences with SMC1–6 from *Saccharomyces cerevisiae* (Sc; NCBI Entrez Accession No. P32908, P38989, P47037, Q12267, Q08204, Q12749), *Arabidopsis thaliana* (At; NP_191027.2, NP_201047.1, NP_180285.2, BAB10693.1, CAC01791.1, BAB11444.1), *Drosophila melanogaster* (Dm; AAM50005.1, AAD52673.1, NP_523374.2, NP_723996.1, Q8T386, AAF56254.1) and *Homo sapiens* (Hs; Q14683, O95347, NP_005436.1, Q9NTJ3, Q8IY18, Q96SB8). Bootstrap values from 500 trees are indicated.

To identify proteins with a function in chromosome organization in trypanosomatids, the genomes of *T. brucei* (Berriman *et al*., 2005) and *Leishmania major* (Ivens *et al*., 2005) were searched for components of SMC complexes. Iterative profile-based searches (Devaux *et al*., 2007) identified candidate orthologues (Fig. 1A) and phylogenetic analysis clarified their relationship with the well-characterized SMC proteins of yeast and animals and plants. Four SMC homologues were found in both genomes and were most closely related to SMC1, SMC2, SMC3 and SMC4, components of cohesin and condensin (Fig. 1B). The non-SMC subunits of the core cohesin and condensin complexes were also present in both genomes (Fig. 1A). Some organisms have evolved meiosis-specific α-kleisin and STAG domain proteins, exemplified by the *Schizosaccharomyces pombe* proteins Rec8 and Rec11 (Ellermeier and Smith, 2005). No candidate genes for meiosis-specific cohesin proteins were identified in the kinetoplastid genomes. No components of other SMC complexes were detected. In particular no SMC5 or SMC6 orthologues and no homologues of any of the known subunits of the SMC5/6 complex (Nse1–6) were identified in trypanosomes.

### Expression profile of TbSCC1 in bloodstream-form trypanosomes

**Fig. 2:**
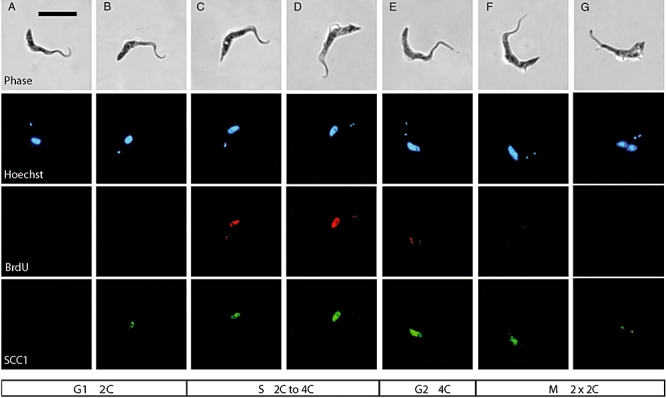
Expression profile of TbSCC1 in cultured bloodstream-form *T. brucei*. The cells were incubated with 5-bromo-2′-deoxyuridine (BrdU) and deoxycytidine prior to immunofluorescent detection of BrdU and SCC1 with appropriate antibodies (Sharma *et al.*, 2008). Panels A–G show cells at different stages of the cell cycle, progressing from G1 through S, G2 and M as indicated. Scale bar represents 10 µm.

In yeast, the expression of SCC1 is regulated during the cell cycle; it is first expressed around the beginning of S-phase and is degraded at anaphase. The expression of SCC1 during the *T. brucei* cell cycle was determined by immunofluorescence labelling of bloodstream forms using an antibody raised against recombinant trypanosome SCC1 (Sharma *et al*., 2008). Trypanosomes have a single mitochondrion with an unusually structured genome, the kinetoplast, which contains a network of concatenated, tightly packed circular DNA molecules. Replication of the kinetoplast occurs in defined phases, in precise temporal co-ordination with the replication of the nuclear genome (Sherwin and Gull, 1989; Woodward and Gull, 1990). The number and relative positions of kinetoplasts (K) and nuclei (N) were used to stage cells within the cell cycle and 5-bromodeoxyuridine (BrdU) incorporation was used as an additional marker for S-phase. Nearly all trypanosomes in G1 (1K1N) showed no SCC1 labelling and no BrdU incorporation (Fig. 2A). Low levels of SCC1 were detected in some 1K1N cells in the absence of detectable BrdU incorporation (Fig. 2B); this may represent cells about to enter S-phase. The intensity of SCC1 staining increased with progression of DNA synthesis as judged by BrdU incorporation (Fig. 2C and D). Cells in G2 (2K1N) showed a strong SCC1 signal, some cells had also incorporated a small amount of BrdU (Fig. 2E), and these cells probably represent cells in the very last stages of S-phase when the labelling period started. The SCC1 expression pattern in bloodstream forms closely resembles that in procyclic forms (Sharma *et al*., 2008).

The antibodies were used to quantify the average number of SCC1 molecules per cell using Western blotting of cell lysates and serial dilutions of known quantities of recombinant TbSCC1 (Sharma *et al*., 2008 and data not shown). The estimate was that lysates from 1 × 10^6^ trypanosomes contain 2.5 ng of SCC1 protein. Taking into consideration the cell cycle-dependent expression pattern of SCC1 described above, and assuming that 40% of cells in an asynchronous culture of procyclic *T. brucei* are in nuclear S- or G2-phase (Woodward and Gull, 1990), these cells contain approximately one SCC1 protein per 1 × 10^3^ bp of DNA.

### SCC1 is essential for normal karyokinesis

**Fig. 3:**
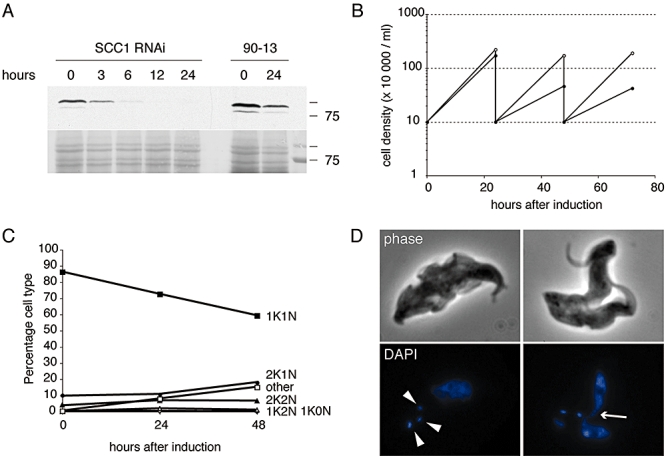
The effect of SCC1 RNAi in bloodstream-form *T. brucei*. A. Detection of SCC1 protein with anti-SCC1C antibodies in Western blots of whole-cell lysates taken 0, 3, 6, 12 and 24 h after addition of doxycycline and control samples from the parental cell line 90-13. The bottom panel shows Ponceau staining of total protein prior to antibody detection. B. Induced cells (filled circles) have a slower growth rate than non-induced controls (open circles). C. SCC1 RNAi leads to a decrease in 1K1N cells and accumulation of cells with abnormal K and N numbers (‘other’). D. Images of DAPI-stained cells viewed by phase-contrast and fluorescence microscopy show the effect of SCC1 RNAi on cellular morphology and on the nucleus and kinetoplast. Multiple kinetoplasts are indicated by arrowheads in the left panel. A typical example of a bilobed nucleus where masses of DNA are connected by thin DNA threads is marked with an arrow in the right panel.

In yeast, the absence of functional SCC1 leads to a failure in sister chromatid cohesion and a loss of viability (Uhlmann *et al*., 1999). To determine if SCC1 had an essential role in trypanosome mitosis, bloodstream forms were transfected with a construct for tetracycline-inducible RNAi based on p2T7-177 (Wickstead *et al*., 2002). By 6 h after induction of RNAi, SCC1 protein was reduced in the induced population, and remained below the detection limit between 12 and 48 h after induction (Fig. 3A and data not shown). Induction of SCC1 RNAi led to a slower population growth rate (Fig. 3B), and the calculated generation time increased from 5.6 h in uninduced cultures to ∼11 h on the second and third day after induction of RNAi. Cells proliferated throughout the experiment and complete growth arrest was not observed.

**Fig. 4:**
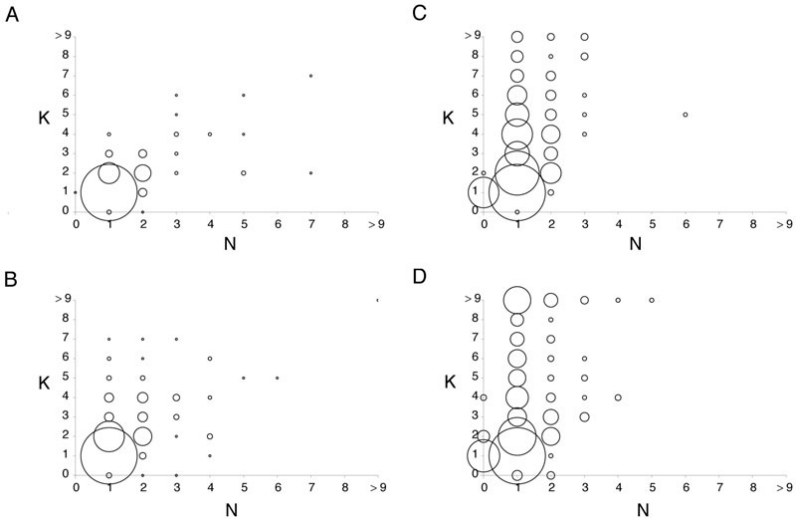
Detailed analysis of kinetoplast (K) and nucleus (N) numbers in bloodstream-form *T. brucei* after induction of SCC1 RNAi (A, 24 h post induction; B, 48 h post induction) and in cells expressing non-cleavable SCC1-mutAB (C, 24 h post induction; D, 48 h post induction). The number of K and N was counted in DAPI-stained cells and plotted. The proportion of cells in each category is represented by the area of the circle. At least 500 cells were scored at each time point.

Induction of SCC1 RNAi resulted in an accumulation of cells with abnormal K and N numbers and multiple flagella. There was a decrease in the fraction of 1K1N cells in the population (Fig. 3C). By 48 h post induction, 13% of cells had more than two kinetoplasts, and 4% of cells had more than two nuclei (Fig. 4). About a quarter of all cells in the population had nuclei with abnormal morphology evident by examination of 4,6-diamidino-2-phenylindole (DAPI)-stained cells. This included cells with normal combinations of K and N numbers. Typically, such aberrant nuclei appeared multi-lobed, with masses of DNA connected by thin DNA bridges (Fig. 3D).

The RNAi phenotype was transient and the growth rate and morphology of cells returned to normal in cultures induced for more than 3 days. This loss of phenotype correlated with a re-appearance of SCC1 protein in Western blots between 48 and 64 h after induction (data not shown) and is most likely due to outgrowth of cells refractory to RNAi.

### Expression of a dominant negative SCC1

**Fig. 5:**
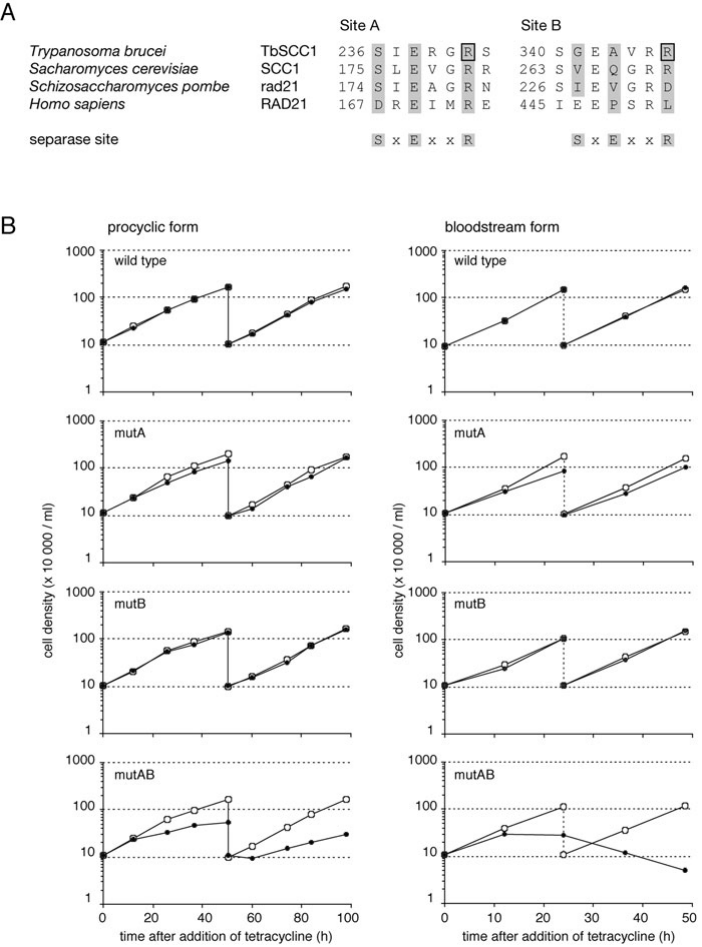
A. Identification of putative separase cleavage sites in TbSCC1 by homology with characterized sites from proteins in other organisms. The residues in the consensus motif are shaded grey and the two arginine residues mutated to aspartic acid in TbSCC1 are boxed. B. The effect of ectopic expression of wild-type SCC1 and separase cleavage site mutants on the growth of *T. brucei* procyclic forms (left) and bloodstream forms (right). Gene expression was induced with tetracycline and the cell density in induced cultures (filled circles) and non-induced controls (open circles) was measured at regular intervals.

Proteolytic cleavage of SCC1 is sufficient to start anaphase in yeast whereas inability to cleave SCC1 results in a failure to separate sister chromatids and an anaphase arrest (Uhlmann *et al*., 1999). Separase is specific for a conserved sequence SxExxRx present once or twice, sites A and B, in all SCC1 orthologues, site A being present in all SCC1 homologues whereas site B is not universally conserved. In the trypanosomatids, *T. brucei* SCC1 contains sites A and B (Fig. 5A) whereas the *L. major* protein only contains site A. Three mutant versions of SCC1 were made with a R → D mutation in separase cleavage site A (mutA) or B (mutB) or both A and B (mutAB). The SCC1 transgenes were then expressed with C-terminal eYFP fusions from a tetracycline-regulated promoter from integrated constructs. Expression of the SCC1 transgenes was used to test whether the mechanism of SCC1 cleavage is conserved and as a tool to study the cellular phenotype of an anaphase arrest caused by inability to cleave SCC1.

The effect of expression of the SCC1 transgenes on growth rate was determined. The wild-type SCC1-eYFP had no effect on growth (Fig. 5B). Expression of SCC1-mutA caused a slight reduction in population growth rate in both bloodstream forms and procyclic forms. Expression of SCC1-mutB had no effect on the growth rate. In contrast, expression of SCC1-mutAB caused a reduction in the growth rate of procyclic forms from 12 h post induction. By 50 h, the reduction in growth was 70% compared with wild type (Fig. 5B). The effect of the double mutation was more pronounced in bloodstream forms, where cell proliferation stopped almost completely at 12 h post induction. From 24 h post induction, cell numbers decreased, indicating cell death (Fig. 5B). Similar results were obtained in separate experiments using SCC1 transgenes with a C-terminal tandem triple myc epitope tag instead of eYFP (data not shown).

**Fig. 6:**
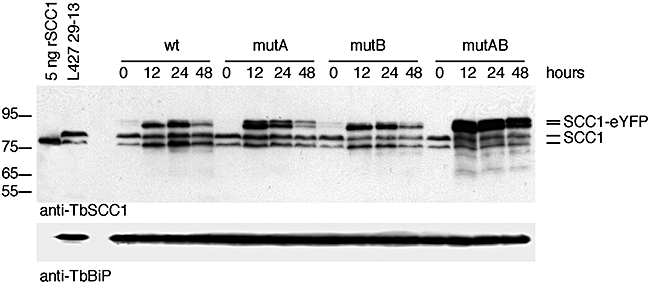
Western blot analysis of a time-course after induction of expression of wild-type and separase cleavage site mutants of TbSCC1 with a C-terminal eYFP tag in procyclic-form *T. brucei* Lister 427 29-13. rSCC1, recombinant TbSCC1 (Sharma *et al*., 2008); L427 29-13, parental host strain Lister 427 29-13 (Wirtz *et al*., 1999). The same samples were probed with anti-BiP as a loading control.

Western blot analysis of whole-cell lysates over a time-course after induction of the transgenes showed that expression of SCC1 peaked at 12–24 h, and that the steady-state levels of SCC1-mutAB were higher than wild type, SCC1-mutA or SCC1-mutB (Fig. 6). The higher levels of SCC1-mutAB were probably due to accumulation of uncleaved protein. The simplest interpretation for the phenotype of SCC1 transgene expression is that mutation of both the potential separase cleavage sites in SCC1 has indeed rendered SCC1-mutAB resistant to cleavage and this in turn resulted in a slowing or cessation of population growth.

On a Western blot, the anti-SCC1 antibody detects a doublet of ∼75 kDa in *T. brucei* whole-cell extracts and a single band at ∼75 kDa when tested against recombinant SCC1 (Fig. 6). The most likely explanation for this is that the lower band in the doublet represents unmodified TbSCC1, while the higher band is a phosphorylated or otherwise modified version. RNAi affects both bands in the doublet (Fig. 3) indicating that these proteins are products of the same gene rather than different proteins that share common epitopes. The idea that TbSCC1 is modified *in vivo* is further supported by the fact that all of the tagged versions of SCC1 examined also run as doublets in Western blots (Fig. 6 and data not shown). In yeast SCC1 is known to be phosphorylated at several residues and phosphorylation of serine residues near the separase cleavage sites are required for efficient cleavage of SCC1 by separase (Alexandru *et al*., 2001). There are serine residues in equivalent positions in TbSCC1.

### The cellular phenotype in procyclic and bloodstream forms

**Fig. 7:**
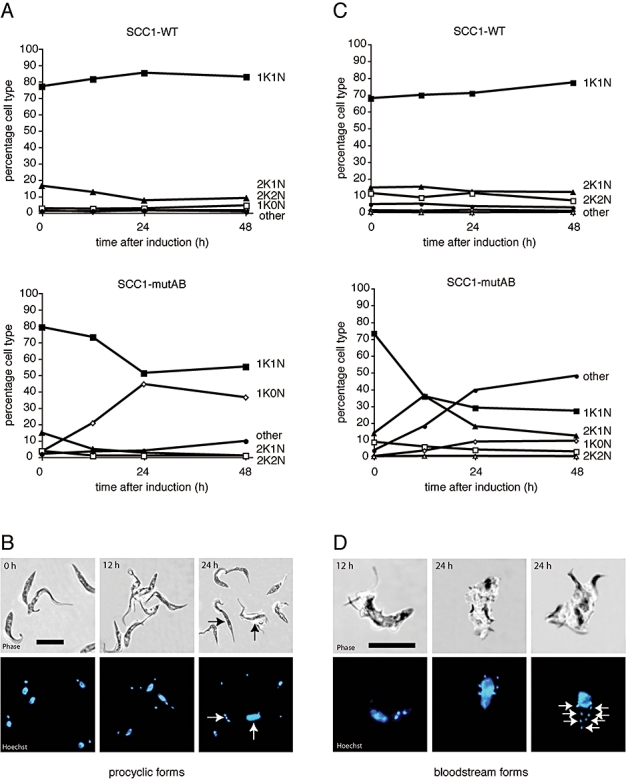
A. Expression of the separase-resistant SCC1 (SCC1-mutAB) in procyclic forms causes a rapid accumulation of anucleate 1K0N cells (zoids). B. The effect of SCC1-mutAB expression on cellular morphology and on the nucleus and kinetoplast in procyclic forms at 0, 12 and 24 h after induction; the arrows indicate one cell with three kinetoplasts and no nucleus and another cell with an enlarged nucleus. C. Expression of SCC1-mutAB in bloodstream forms causes a rapid accumulation of cells with multiple kinetoplasts (category ‘others’) and yields a small number of zoids. Filled square: 1K1N; filled triangle: 2K1N; open square: 2K2N; open diamond: 1K0N (zoids); open triangle: 1K2N; filled circle: ‘others’. D. Bloodstream forms at 12 and 24 h after induction; the arrows indicate the multiple kinetoplasts in a typical cell.

Expression of SCC1 transgenes was induced and the nuclear and kinetoplast DNA visualized in individual cells over a time-course. Cells were categorized according to the number of kinetoplasts (K) and nuclei (N) to determine cell cycle stage (Woodward and Gull, 1990). In populations of procyclic trypanosomes expressing wild-type SCC1, SCC1-mutA or SCC1-mutB, ∼80% of cells were 1K1N in G1 with 8–17% in S-phase. Zoids, which are anucleate cells with one kinetoplast, 1K0N, formed when cytokinesis occurs in the absence of mitosis (Robinson *et al*., 1995), were rare. In contrast, procyclic trypanosomes expressing SCC1-mutAB produced large numbers of zoids (20% at 12 h, 44% at 24 h and 36% at 48 h post induction) (Fig. 7A). In addition, 9% of cells expressing the SCC1-mutAB had abnormal combinations of K and N numbers and some cells had enlarged nuclei (Fig. 7B).

Bloodstream trypanosomes expressing wild-type SCC1, SCC1-mutA or SCC1-mutB were also similar to wild-type cells. Expression of SCC1-mutAB caused a sharp decrease in the proportion of cells with 1K1N, from 74% in uninduced populations to 30% at 24 h after induction. Cells with multiple kinetoplasts accumulated rapidly. At 12 h after induction the proportion of 2K1N cells had increased from 14% to 36%. By 48 h after induction, more than half of the cells in the population had abnormal combinations of K and N numbers (Figs 4C and D and 7C). The number of bloodstream-form zoids was small (9%).

Microscopic examination of DAPI-stained bloodstream forms expressing SCC1-mutAB showed multiple morphological abnormalities, with a single enlarged nucleus, multiple kinetoplasts and multiple flagella (Fig. 7D). The number of the kinetoplasts and flagella in individual cells indicated that new rounds of organelle duplication had occurred after a failed mitosis.

In both bloodstream-form and procyclic trypanosomes the result of SCC1-mutAB expression was a failure in nuclear division with no indication of progression beyond anaphase A. In procyclic forms cytokinesis still occurred and a zoid was produced; in bloodstream forms cell division was not successfully completed.

**Fig. 8:**
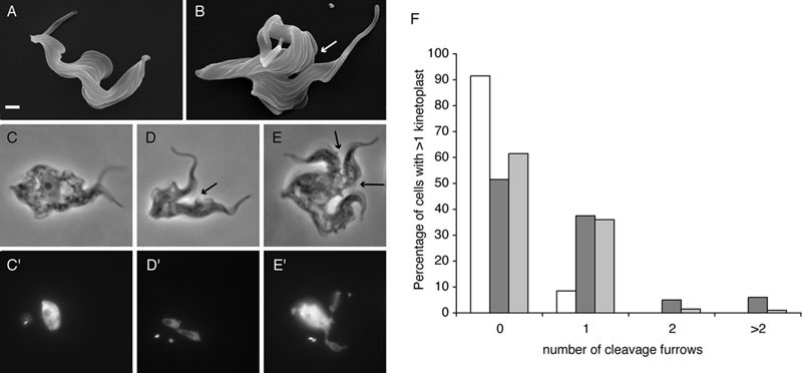
Bloodstream-form *T. brucei* expressing SCC1-mutAB and cells induced for SCC1 RNAi fail to complete cytokinesis. A and B. (A) Morphology of a bloodstream-form trypanosome viewed by scanning electron microscopy before induction of SCC1-mutAB expression; (B) 12 h after induction. The arrow indicates a cleavage furrow. Scale bar represents 1 μm. C–E. Phase-contrast images of cells with zero, one or two cleavage furrows, respectively, indicated by arrows. (C′)–(E′) show the DAPI-stained DNA in these cells. Images were taken 24 h after induction of SCC1-mutAB expression. F. At 24 h post induction, cells with more than one kinetoplast were examined for the presence of cleavage furrows. The graph shows the proportion of cells with zero, one or two cleavage furrows. White bars: SCC1-mutAB-uninduced control; dark grey bars: SCC1-mutAB; light grey bars: SCC1 RNAi.

**Fig. 9:**
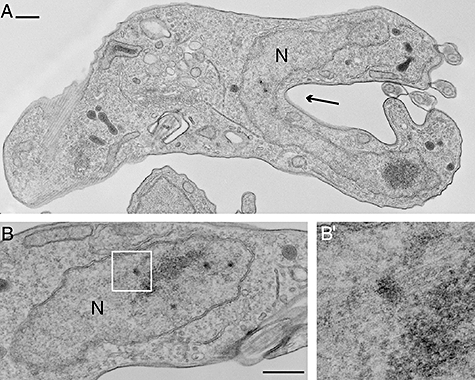
Transmission electron microscopy shows the effect of expression of SCC1-mutAB on nuclear ultrastructure of bloodstream forms. The cell in (A) has a cleavage furrow (arrow) in close apposition to the undivided nucleus (N). Electron-dense plaques are visible in the nucleus in (A) and (B). The association of plaques with spindle microtubules can be seen in (B). (B′) shows the boxed area at a higher magnification. Scale bars represent 500 nm.

Examination of bloodstream-form trypanosomes expressing SCC1-mutAB by light and electron microscopy revealed that many had prominent cleavage furrows (Figs 8 and 9) and thus population growth arrest (Fig. 5B) occurred due to a failure to complete cell division as opposed to a failure to initiate cell division. At 24 h post induction, 38% of cells with more than one kinetoplast had one cleavage furrow and 11% had two or more (Fig. 8F). SCC1 knockdown by RNAi also resulted in cells with arrested cleavage furrows; 36% of cells with more than one kinetoplast had one cleavage furrow and 3% had two or more (Fig. 8F).

The posterior end of the stalled cleavage furrow was often in close apposition with the undivided multi-lobed nucleus (Fig. 8D and E; see also Fig. 3D). The proximity of the stalled furrow to the nucleus is illustrated at higher resolution in a thin section through a cell expressing SCC1-mutAB (Fig. 9). These observations suggest that, in bloodstream forms, the undivided nucleus may form a physical obstacle to further cleavage furrow ingression.

After both expression of SCC1-mutAB and induction of SCC1 knockdown the cell nuclei are enlarged and irregularly shaped. Examination by transmission electron microscopy shows distinct abnormalities in nuclear ultrastructure. Electron-dense plaques are frequently observed (Fig. 9), often associated with spindle microtubules (Fig. 9B). Many nuclei are no longer of a regular oval shape, but instead have multiple protrusions and invaginations of the nuclear envelope.

Taken together, these results provide evidence that the gene Tb927.7.6900 identified as the closest homologue of yeast SCC1 is indeed the functional orthologue. *T. brucei* is the most divergent eukaryote for which the mechanism of sister chromatid separation by SCC1 cleavage by separase has been demonstrated. The consequences of expression of a separase-resistant form of SCC1 were an arrest of mitosis prior to separation of sister chromatids. Mitotic arrest resulted in different cellular phenotypes in two life cycle stages; bloodstream forms initiated cytokinesis without progressing to abscission, while procyclic forms completed cytokinesis and produced zoids.

## Discussion

The main findings of this study are: (i) the *T. brucei* genome contains homologues of all the components present in cohesin and condensin, two SMC complexes necessary for the correct segregation of chromosomes at mitosis, (ii) SCC1, the kleisin component of cohesin, is expressed from the beginning of S-phase until anaphase, (iii) knockdown of expression of SCC1 by RNAi impedes nuclear division, (iv) expression of a dominant negative mutant of SCC1 that is resistant to cleavage by separase blocks nuclear division, and (v) reduction of wild-type SCC1 activity or expression of separase-resistant SCC1 resulted in a failure to complete cell division in bloodstream forms but not in procyclic forms which divided to produce one nucleated and one anucleate daughter cell.

Cohesin is the complex that physically holds the two sister chromatids together from S-phase to anaphase.Proteolytic cleavage of the SCC1 subunit by separase at one of two specific sites is essential for sister chromatid separation at anaphase. The results from this study provide evidence that the gene Tb927.7.6900 identified as the closest homologue to yeast SCC1 is the functional orthologue; the expression pattern and nuclear phenotype of both the knockdown and dominant negative are similar to equivalent mutations in the SCC1 genes from yeast and *Drosophila* (Uhlmann *et al*., 1999; Vass *et al*., 2003). Knockdown of TbSCC1 by RNAi in bloodstream-form trypanosomes resulted in a phenotype consistent with a delayed progression through the cell cycle and aberrant segregation of nuclear DNA. Expression of non-cleavable SCC1-mutAB produced a more severe phenotype, characterized in bloodstream-form trypanosomes by a growth arrest at 12 h post induction and a decrease of cell numbers 24 h post induction indicating cell death. During mitosis, individual *T. brucei* chromosomes do not visibly condense but the movement of DNA clusters to opposite poles of the nucleus follows a defined pattern that can be observed in DAPI-stained cells (Ogbadoyi *et al*., 2000). The sharp increase in the proportion of cells with 2K1N early after induction of SCC1-mutAB expression, and accumulation of cells with > 2K1N indicate a block of nuclear division early in mitosis. This phenotype is consistent with a block in sister chromatid segregation resulting in failure of anaphase A and the absence of anaphase B events including spindle elongation and correct nuclear positioning. Expression of separase-resistant SCC1 prevented both procyclic and bloodstream-form trypanosomes from partitioning nuclear DNA into two clusters of equal size and no late mitotic, anaphase B, nuclei were observed. The inducible expression of SCC1 with mutations in both separase cleavage sites (SCC1-mutAB) thus provided an experimental tool to investigate the phenotype of cells with nuclei unable to separate sister chromatids during mitosis. The conservation of function of SCC1, the kleisin subunit of cohesin and the presence of homologues for the other cohesin subunits in the genome provide evidence for a conserved mechanism of sister chromatid cohesion common to all eukaryotes. The mutational analysis of the separase cleavage sites showed that one functional cleavage site is necessary and sufficient for normal progression of *T. brucei* mitosis, a similar finding was made in yeast (Uhlmann *et al*., 2000). Separase cleavage of SCC1 as a trigger for anaphase was first discovered in the budding yeast *Saccharomyces cerevisiae* (Uhlmann *et al*., 1999; 2000) and subsequently in metazoa (Hauf *et al*., 2001; Jager *et al*., 2001; Wirth *et al*., 2006). Our data show that the mechanism whereby separase cleaves SCC1 to release chromosome cohesion evolved early in eukaryotic evolution, before the eugleonozoan and animal, fungal and plant lineages diverged, possibly almost two billion years ago (Hedges *et al*., 2004).

Entry into mitosis and progression to metaphase is dependent on the activity of the cyclin-dependent kinase CDK1 in all eukaryotes studied to date. Progression from metaphase to anaphase is dependent on activation of the anaphase-promoting complex or cyclosome (APC/C). The APC/C is the E3 ubiquitin ligase that indirectly activates the protease separase by targeting its inhibitory subunit securin for degradation (de Gramont and Cohen-Fix, 2005). Separase is then responsible for cleavage of SCC1, permitting anaphase, and independently initiates the release of the CDC14 phosphatase from the nucleolus (Shou *et al*., 1999), which is necessary for mitotic exit and probably reverses the CDK1 phosphorylation. This simplified pathway was elucidated in yeast and, although all steps have not been confirmed in trypanosomes, orthologues of CDK1, cyclin and the APC/C have been identified and homologues of separase (Tb927.1.3120) (Mottram *et al*., 2003) and CDC14 (Tb11.01.4270) are encoded in the trypanosomatid genomes. In *T. brucei,* CYC6/CRK3 are the orthologues of cyclin B/CDK1. Depletion of CYC6 by RNAi results in a mitotic block; in procyclic forms, cell division occurs in the absence of nuclear division producing a zoid daughter cell whereas in bloodstream forms there is an accumulation of cells with a single enlarged nucleus and multiple kinetoplasts (Hammarton *et al*., 2003; Tu and Wang, 2004). Similar experiments with CRK3 produced a similar phenotype (Kumar and Wang, 2005). The different phenotypes in procyclic and bloodstream forms led to the suggestion that regulation of cell cycle progression differs in the two life cycle stages and that a mitosis to cytokinesis checkpoint only operates in bloodstream forms (Ploubidou *et al*., 1999; Hammarton *et al*., 2003; Tu and Wang, 2005). The depletion of APC/C activity through RNAi knockdown of CDC27 or APC1 resulted in cell populations enriched in G2/M cells; in procyclic forms cells arrested with two kinetoplasts and a single enlarged nucleus containing a short spindle whereas bloodstream-form cells arrested with two kinetoplasts and a nucleus arrested in late anaphase (Kumar and Wang, 2005).

In procyclic forms, the phenotype of cells expressing separase-resistant SCC1 was similar to cells in which CRK3 or CYC6 had been depleted with the production of zoids resulting from cell division in the absence of nuclear division. The accumulation of zoids in cultures expressing separase-resistant SCC1 (40% after 24 h) was similar both to that obtained using the microtubule assembly inhibitor rhizoxin (30% zoids after 8 h) (Robinson *et al*., 1995; Ploubidou *et al*., 1999) and to that obtained after RNAi depletion of CYC6 (∼40% after 48 h) (Hammarton *et al*., 2003). In contrast, APC/C-depleted procyclic forms accumulated as 2K1N cells, the nucleus containing a short spindle consistent with an anaphase A arrest, but did not undergo cytokinesis.

Bloodstream-form cells expressing separase-resistant SCC1 had a different phenotype to CRK3- or CYC6-depleted cells. In both cases, cells arrested with an enlarged nucleus and multiple kinetoplasts but cells expressing separase-resistant SCC1 initiated cytokinesis whereas cells depleted of CRK3 and CYC6 did not. Depletion of APC/C activity caused a late anaphase arrest with an elongated spindle. The bloodstream-form phenotype observed in our study also differed markedly from the very specific precytokinesis cell cycle arrest observed when variant surface glycoprotein (VSG) transcripts were ablated by RNAi (Sheader *et al*., 2005). VSG RNAi caused a rapid accumulation of 2K2N cells with two external flagella and, in a minority of cells, additional internal flagella. No internal flagella were observed in SCC1 mutants. Clearly, not all bloodstream-form cytokinesis defects are the same and specific phenotype patterns are now emerging.

Previous RNAi studies manipulated CDK1 (CYC6/CRK3) and APC/C activity; both are regulators of the cell cycle and knockdown will have pleiotropic effects; for example, a reduction of CDK1 activity will not only affect phosphorylation of its substrates but also the substrates of downstream kinases and knockdown of the APC/C components will reduce separase activation which will prevent cleavage of SCC1 but also block the role of separase in the activation of CDC14 and mitotic exit. In contrast, SCC1 is a structural component with a single role in sister chromatid cohesion and is a substrate for cell cycle regulators. Manipulation of SCC1 was used to reveal the phenotype resulting from defects in sister chromatid cohesion. RNAi knockdown of SCC1 prevented a normal mitosis and expression of the separase-resistant SCC1 allowed an analysis of the phenotype of an early anaphase block. In both of the life cycle stages investigated cytokinesis was initiated in the presence of separase-resistant SCC1 or after SCC1 knockdown. Thus, the initiation of cytokinesis is not dependent on the completion of mitosis and there is no checkpoint capable of blocking initiation of cell division in response to incomplete mitosis in either life cycle stage. In *T. brucei* procyclic forms, complete cytokinesis occurred in the absence of nuclear division whereas bloodstream forms arrested with stalled cleavage furrows.

Why is cytokinesis in bloodstream-form cells incomplete after expression of separase-resistant SCC1? The trypanosome cytokinesis machinery and mechanisms of its activation are unknown (Hammarton *et al*., 2007) so it is possible in bloodstream forms that furrow ingression was blocked by a checkpoint mechanism in response to a signal indicating that mitosis had failed but this would be without precedent. Alternatively, the architecture of the bloodstream trypanosome may physically prevent cytokinesis in the SCC1 mutants. In procyclic trypanosomes, the position of the anterior nucleus remains fixed, through an as yet uncharacterized anchoring system, whereas the posterior nucleus ‘moves’ into the gap between the segregated basal bodies (Robinson *et al*., 1995). As the kinetoplast is physically linked to the basal body, prior to cell division the order of organelles from the anterior end is alternating kinetoplasts and nuclei – KNKN (Robinson *et al*., 1995). If the posterior daughter cell receives no nucleus then a 1K0N zoid and a 1K1N* cell (N* denotes a nucleus with replicated but non-segregated DNA) are produced by cell division, as observed when separase-resistant SCC1 was expressed. Prior to cell division, the relative positions of nuclei and kinetoplasts differ between bloodstream and procyclic forms; the order is KKNN in bloodstream forms and KNKN in procyclic forms (Tyler *et al*., 2001). In cells expressing separase-resistant SCC1, the close apposition of the partial cleavage furrows with the nuclear membrane evident in the electron micrographs suggests that an undivided nucleus may form a physical barrier that prevents, or slows down, further cleavage furrow ingression. Thus, the different outcomes of separase-resistant SCC1 expression in the two life cycle stages investigated can be explained by the different geometry of organelle position in the two cell types.

The difference between the separase-resistant SCC1 dominant negative phenotype and the CYC6 and CRK3 knockdown phenotypes is informative. In procyclic forms, the phenotypes were similar whereas in bloodstream forms no initiation of cleavage was reported when CYC6 or CRK3 was depleted. One possible interpretation is that CYC6/CRK3 was required for initiation of cleavage in bloodstream forms, possibly through activation of the APC/C, but not in procyclic forms. However it would be worth confirming first the precise degree of CRK3 or CYC6 knockdown and whether there was any effect on APC/C activation. The absence of cell division in procyclic cells with depleted APC/C activity cannot be explained solely by a failure to licence SCC1 cleavage but suggests the APC/C activity may be required for initiation of cell division, possibly through its role in the activation of CDC14 as occurs in yeast. In bloodstream forms, depletion of APC/C led to a late anaphase arrest whereas expression of separase-resistant SCC1 resulted in an arrest in early anaphase with no elongation of the nucleus. The easiest explanation for these contrasting observations is that the depletion of APC/C was not complete but was insufficient to activate the mitotic exit network (Sullivan and Morgan, 2007). In yeast mutation in mitotic exit network genes results in a late anaphase arrest (Jaspersen *et al*., 1998).

The experiments presented here provide no evidence for checkpoints that link the completion of mitosis to cytokinesis initiation in the two *T. brucei* life cycle stages investigated. Organelle duplication proceeds in the absence of a successful mitosis as several rounds of basal body and kinetoplast duplication occurred in bloodstream forms although not in procyclic forms. Re-initiation of nuclear S-phase was not directly determined but the nuclei were enlarged in procyclic and bloodstream forms after expression of separase-resistant SCC1 suggesting endoreduplication was occurring.

The results of the experiments above show that in the two *T. brucei* life cycle stages investigated, initiation of cytokinesis is independent of sister chromatid cohesion or cohesin release following SCC1 cleavage. The nuclear division defect caused by expressing non-cleavable TbSCC1 is reminiscent of the phenotypes of yeast and human cells that express non-cleavable SCC1. In *S. cerevisiae*, cytokinesis was delayed but not inhibited and progeny with abnormal DNA content were produced (Uhlmann *et al*., 1999). In human cells, cytokinesis was initiated but not completed and sister chromatid separation was shown not to be required for cyclin B destruction or mitotic exit (Hauf *et al*., 2001). Depletion of TbSCC1 by RNAi in bloodstream forms led to slower proliferation without cell cycle arrest and partial ingression of cleavage furrows. In yeast and higher eukaryotes a spindle assembly checkpoint inhibits APC/C and prevents anaphase onset until tension generated by sister chromatid cohesion and bipolar attachment to the spindle microtubules is sensed. With the exception of Mad2p, no homologues of known spindle checkpoint proteins were found in the trypanosome genome (Berriman *et al*., 2005). Progression of the cell cycle in the absence of SCC1 could indicate that such a checkpoint is absent or, alternatively, that an arrest is only transient.

## Experimental procedures

### Bioinformatics

Kinetoplastid genome sequences were accessed via GeneDB (http://www.genedb.org/). Iterative profile-based searches (Devaux *et al*., 2007) were used to identify sequences in the kinetoplastid genomes. Seed alignments used to generate hidden Markov models (Eddy, 1998) were produced from sequences identified with simple blast searches and Pfam alignments (Bateman *et al*., 2004). For phylogenetic reconstruction, SMC protein sequences were aligned in clustal x, manually edited and a maximum parsimony tree was built with paup* 4.0b10 (Swofford, 1998).

### Cells

*Trypanosoma brucei brucei* Lister 427-derived bloodstream-form cell line 90-13 (Wirtz *et al*., 1999) was cultured in HMI-9 (Hirumi and Hirumi, 1994) and procyclic cell line 29-13 (Wirtz *et al*., 1999) was grown in SDM-79 (Brun and Schonenberger, 1979).

### Production of antibody and immunofluorescence microscopy

To produce anti-SCC1C, the 5′ end of the TbSCC1 coding sequence (encoding amino acids 390–584) was amplified with primers 5′-GACGGATCCCCGCTCGCAAGGG-3′ and 5′-AAGCTTACACAGTGAGTTGCACCTC-3′ and cloned in the BamHI and HindIII sites of pQE-30 (Qiagen) to generate an in-frame fusion with a 6xHistidine tag. The protein was purified from *Escherichia coli* M15 pREP4 on Ni-NTA superflow resin (Qiagen) under denaturing conditions, precipitated with acetone and used to immunize rabbits. Immunofluorescence detection of SCC1 and BrdU was performed as previously described (Sharma *et al*., 2008).

For generation of the RNAi cell line, a 536 bp fragment of TbSCC1 was PCR-amplified from genomic DNA using primers 5′-TCTAGACCTTTTCTCTCCGCTTACG-3′ and 5′-TCTAGACTCCATTTCTTCACGGTCAC-3′, digested with XbaI and cloned in the XbaI site of p2T7-177 (Wickstead *et al*., 2002). The resulting plasmid p2T7-177-SCC1 was linearized with NotI and used to transfect bloodstream-form 90-13 cells by electroporation. Transfected cells were selected with 2.5 μg ml^−1^ Phleomycin and RNAi was induced using 1 μg ml^−1^ doxycycline.

Cell growth was monitored using a CASY cell-counter and analyser system (Schärfe System GmbH). For DNA staining, cells were spread onto glass slides, fixed in 2–4% paraformaldehyde in phosphate-buffered saline (PBS) and embedded in Vectashield (Vector Laboratories) with DAPI. Images were captured on a Leitz DMRB fluorescence microscope (Leica Microsystems) using a Coolsnap FX Camera (Photometrics) and assembled in Adobe Photoshop.

Site-directed mutagenesis of SCC1 to produce SCC1-mutA, mutB, mutAB was carried out using standard procedures. All transgenes were fully sequenced after mutagenesis. Expression used either p2216 or p2280 (Kelly *et al*., 2007) in the procyclic-form cell line Lister 427 pLEW29 pLEW13 or the bloodstream-form cell line Lister 427 pLEW90 pLEW13 (Wirtz *et al*., 1999).

### Electron microscopy

Bloodstream-form cells were fixed in 2.5% glutaraldehyde, 2% formaldehyde, 0.1% picric acid in 100 mM phosphate buffer pH 7.0 for 2 h. For thin-section electron microscopy, cells were then processed as described in Dawe *et al*. (2005). For scanning electron microscopy, fixed cells were left to adhere to glass coverslips and processed as described in Sharma *et al*. (2008).
